# Multiple Solutions for a Singular Quasilinear Elliptic System

**DOI:** 10.1155/2013/278013

**Published:** 2013-10-24

**Authors:** Lin Chen, Caisheng Chen, Zonghu Xiu

**Affiliations:** ^1^College of Science, Hohai University, Nanjing 210098, China; ^2^College of Mathematics and Statistics, Yili Normal University, Yining 835000, China; ^3^Science and Information College, Qingdao Agricultural University, Qingdao 266109, China

## Abstract

We consider the multiplicity of nontrivial solutions of the following quasilinear elliptic system −div(|*x*|^−*ap*^|∇*u*|^*p*−2^∇*u*) + *f*_1_(*x*)|*u*|^*p*−2^ *u* = (*α*/(*α* + *β*))*g*(*x*)|*u*|^*α*−2^ *u*|*v*|^*β*^ + *λh*_1_(*x*)|*u*|^*γ*−2^ *u* + *l*_1_(*x*), −div(|*x*|^−*ap*^|∇*v*|^*p*−2^∇*v*) + *f*_2_(*x*)|*v*|^*p*−2^ *v* = (*β*/(*α* + *β*))*g*(*x*)|*v*|^*β*−2^ *v*|*u*|^*α*^ + *μh*_2_(*x*)|*v*|^*γ*−2^ *v* + *l*_2_(*x*), *u*(*x*) > 0, *v*(*x*) > 0, *x* ∈ ℝ^*N*^, where *λ*, *μ* > 0, 1 < *p* < *N*, 1 < *γ* < *p* < *α* + *β* < p* = *Np*/(*N* − *pd*), 0 ≤ *a* < (*N* − *p*)/*p*, *a* ≤ *b* < *a* + 1, *d* = *a* + 1 − *b* > 0. The functions *f*_1_(*x*), *f*_2_(*x*), *g*(*x*), *h*_1_(*x*), *h*_2_(*x*), *l*_1_(*x*), and *l*_2_(*x*) satisfy some suitable conditions. We will prove that the problem has at least two nontrivial solutions by using Mountain Pass Theorem and Ekeland's variational principle.

## 1. Introduction

In this paper, we consider the multiplicity of solutions to a class of quasilinear elliptic system

(1) −div⁡(|x|−ap|∇u|p−2∇u)+f1(x)|u|p−2u  =αα+βg(x)|u|α−2u|v|β   +λh1(x)|u|γ−2u+l1(x),−div⁡(|x|−ap|∇v|p−2∇v)+f2(x)|v|p−2v  =βα+βg(x)|v|β−2v|u|α   +μh2(x)|v|γ−2v+l2(x),u(x)>0, v(x)>0, x∈ℝN,

where *λ*, *μ* > 0, 1 < *p* < *N*, 1 < *γ* < *p* < *α* + *β* < p* = *Np*/(*N* − *pd*), 0 ≤ *a* < (*N* − *p*)/*p*, *a* ≤ *b* < *a* + 1, *d* = *a* + 1 − *b* > 0.

In recent 20 years, existence and multiplicity for a quasilinear elliptic equation and equations have been studied widely. In [1], Yu has studied the existence of a nontrivial weak solution to the quasilinear elliptic problem

(2) −div⁡(a(x)|∇u|p−2∇u)+b(x)|u|p−2u  =g(x)|u|r−2u, in  Ω,u=0, on⁡∂Ω,

where *Ω* is a smooth exterior domain in ℝ^*N*^, 1 < *p* < *N*, $a(x)\in L^{\infty }(\Omega )\cap C^{0,\delta }(\overset{-}{\Omega })$ is a positive function, and *b* ∈ *L* ^*∞*^(*Ω*)∩*C*(*Ω*) is a nonnegative function. Problems of this type are motivated by mathematical physics, since certain stationary waves in nonlinear Klein-Gordon or Schrödinger equations can be reduced to this form (see [2, 3]). Problem (2) began to attract more and more attention after the work of Yu [1]; we refer the readers to [4–8].

In [9], Hsu studied the following elliptic system:

(3) $$\begin{array}{c} -\mathrm{div}\left({\left|\nabla u\right|}^{p-2}\nabla u\right)=\lambda {\left|u\right|}^{q-2}u+\frac{2\alpha }{\alpha +\beta }{\left|u\right|}^{\alpha -2}u{\left|v\right|}^{\beta },\;x\in \Omega ,\\ -\mathrm{div}\left({\left|\nabla v\right|}^{p-2}\nabla v\right)=\mu {\left|v\right|}^{q-2}v+\frac{2\beta }{\alpha +\beta }{\left|v\right|}^{\beta -2}v{\left|u\right|}^{\alpha },\;x\in \Omega ,\\ u=v=0,\;x\in \partial \Omega , \end{array}$$

where *Ω* ⊂ ℝ^*N*^ is a bounded domain, *λ*, *μ* > 0, 1 < *p* < *q* < *N*, and *α* > 1, *β* > 1 satisfy *α* + *β* = p*. The problem (3) is the special case of problem (1), while *a* = 0, *f*_1_(*x*) = *f*_2_(*x*) = 0. By applying Nehari manifold method, the author proved the problem (3) has at least one positive solution when 0 < *λ* ^*p*/(*p*−*q*)^ + *μ* ^*p*/(*p*−*q*)^ < *λ*_1_ and has at least two positive solutions when 0 < *λ* ^*p*/(*p*−*q*)^ + *μ* ^*p*/(*p*−*q*)^ < *λ*_2_.

This paper is motivated by the results of [9–12]. Replacing the Nehari manifold methods, we use the Mountain Pass Theory and Ekeland's variational principle to study the existence of multiple solutions for problem (1). It seems difficult to get the same result by Nehari manifold methods.

Throughout this paper, we make the following hypotheses:

(*H*_1_) *h*_*i*_ ∈ *L* ^*∞*^(ℝ^*N*^), *h*_*i*_(*x*)|*x*|^*bγ*^ ∈ *L* ^*θ*^(ℝ^*N*^), *θ* = p*/(p* − *γ*), *i* = 1,2.
(*H*_2_) *g*(*x*)|*x*|^*b*(*α*+*β*)^ ∈ *L* ^*δ*^(ℝ^*N*^)∩*L* ^*∞*^(ℝ^*N*^), *δ* = p*/(p* − *α* − *β*).
(*H*_3_) *l*_1_(*x*)|*x*|^*b*^, *l*_2_(*x*)|*x*|^*b*^ ∈ *L* ^*σ*^(*Ω*), *σ* = p*/(p* − 1), *l*_1_(*x*) > 0, *l*_2_(*x*) > 0.

In this paper, we let *L*_*b*_^*p*^(ℝ^*N*^) the completion of the space *C*_0_^*∞*^(ℝ^*N*^) with respect to the norm ||*u*||_*L*_*b*_^*p*^_ = (∫_ℝ^*N*^_|*x*|^−*bp*^|*u*|^*p*^ *dx*)^1/*p*^, and let *W*_*a*_^1,*p*^(ℝ^*N*^) the completion of *C*_0_^*∞*^(ℝ^*N*^) with respect to the norm ||*u*||_*W*_*a*_^1,*p*^_ = (∫_ℝ^*N*^_ | *x*|^−*ap*^ | ∇*u*|^*p*^ *dx*)^1/*p*^.

We define

(4) $$\begin{array}{c} h_{1\theta }={\left(\int_{{\mathbb{R}}^{N}}{\left(\left|h_{1}\left(x\right)\right|{\left|x\right|}^{b\gamma }\right)}^{\theta }dx\right)}^{1/\theta },\\ h_{2\theta }={\left(\int_{{\mathbb{R}}^{N}}{\left(\left|h_{2}\left(x\right)\right|{\left|x\right|}^{b\gamma }\right)}^{\theta }dx\right)}^{1/\theta },\\ l_{1\sigma }={\left(\int_{{\mathbb{R}}^{N}}{\left|x\right|}^{b\sigma }{\left|l_{1}\left(x\right)\right|}^{\sigma }dx\right)}^{1/\sigma },\\ l_{2\sigma }={\left(\int_{{\mathbb{R}}^{N}}{\left|x\right|}^{b\sigma }{\left|l_{2}\left(x\right)\right|}^{\sigma }dx\right)}^{1/\sigma }, \end{array}$$

where *θ* = p*/(p* − *γ*), *σ* = p*/(p* − 1).

The following inequality is called Caffarelli-Kohn-Nirenberg inequality [13]. There is a constant *S* > 0 such that

(5) $$\begin{array}{c} {\left(\int_{{\mathbb{R}}^{N}}{\left|x\right|}^{-bp^{*}}{\left|u\right|}^{p^{*}}dx\right)}^{1/p^{*}}\leq S{\left(\int_{{\mathbb{R}}^{N}}{\left|x\right|}^{-ap}{\left|\nabla u\right|}^{p}dx\right)}^{1/p}, \end{array}$$

where −*∞* < *a* < (*N* − *p*)/*p*, *a* ≤ *b* < *a* + 1, *d* = *a* + 1 − *b*, and p* = *pN*/(*N* − *pd*).

Let *X* = *W*_*a*_^1,*p*^(ℝ^*N*^) × *W*_*a*_^1,*p*^(ℝ^*N*^) denote the usual Sobolev space with the norm

(6) ||(u,v)||=(∫ℝN|x|−ap|∇u|p+f1(x)|u|p)dx +∫ℝN(|x|−ap|∇v|p+f2(x)|v|pdx)1/p.

Then *X* is a reflexive and separable Banach space endowed with the norm ||(·, ·)||.

**Definition 1**

We say that (*u*, *v*) ∈ *X* is a weak solution of problem (1) if for all (*φ*, *ψ*) ∈ *X*, there holds

(7) ∫ℝN(|x|−ap|∇u|p−2∇u∇φ+f1(x)|u|p−2uφ)dx  +∫ℝN(|x|−ap|∇v|p−2∇v∇ψ+f2(x)|v|p−2vψ)dx  −∫ℝNl1(x)φdx−∫ℝNl2(x)ψdx  −αα+β∫ℝN(g(x)|u|α−2u|v|β+λh1(x)|u|γ−2u)φdx  −βα+β∫ℝN(g(x)|v|β−2v|u|α+μh2(x)|v|γ−2v)ψdx =0.

It is clear that problem (1) has a variational structure. Let *J*_*λ*,*μ*_ : *X* → ℝ^1^ be the energy functional corresponding to problem (1) which is defined by

(8) Jλ,μ(u,v)=1p||(u,v)||p−1α+β∫ℝNg(x)|u|α|v|βdx −1γGλ,μ(u,v)−L(u,v),

where

(9) $$\begin{array}{c} G_{\lambda ,\mu }\left(u,v\right)=\int_{{\mathbb{R}}^{N}}\lambda h_{1}\left(x\right){\left|u\right|}^{\gamma }dx+\int_{{\mathbb{R}}^{N}}\mu h_{2}\left(x\right){\left|v\right|}^{\gamma }dx,\\ L\left(u,v\right)=\int_{{\mathbb{R}}^{N}}\left(l_{1}\left(x\right)u+l_{2}\left(x\right)v\right)dx. \end{array}$$

Then, we see that functional *J*_*λ*,*μ*_ ∈ *C* ^1^(*X*, ℝ^1^) and for all (*φ*, *ψ*) ∈ *X*, there holds

(10) 〈Jλ,μ′(u,v),(φ,ψ)〉=∫ℝN(|x|−ap|∇u|p−2∇u∇φ   +f1(x)|u|p−2uφ)dx+∫ℝN(|x|−ap|∇v|p−2∇v∇ψ    +f2(x)|v|p−2vψ)dx−αα+β∫ℝN(g(x)|u|α−2u|v|β           +λh1(x)|u|γ−2u)φdx−βα+β∫ℝN(g(x)|v|β−2v|u|α          +μh2(x)|v|γ−2v)ψdx−∫ℝNl1(x)φdx−∫ℝNl2(x)ψdx.

In particular, it follows from (10) that

(11) 〈Jλ,μ′(u,v),(u,v)〉=||(u,v)||p−∫ℝNg(x)|u|α|v|βdx−Gλ,μ(u,v)−L(u,v).

It is clear that the critical points of the energy functional *J*_*λ*,*μ*_(*u*, *v*) correspond to the weak solutions of problem (1). Thus, to prove the existence of weak solutions for the problem (1), it is sufficient to show that *J*_*λ*,*μ*_(*u*, *v*) admits a sequence of critical points in *X*. Our main result is the following.

**Theorem 2**

Assume that (*H*_1_)–(*H*_3_) hold. There exist *c*_0_, *c*_1_ > 0 such that if *λ*, *μ* > 0 satisfy 0 < *λh*_1*θ*_ + *μh*_2*θ*_ < *c*_0_ and 0 ≤ *l*_1*σ*_^*p*/(*p*−1)^ + *l*_2*σ*_^*p*/(*p*−1)^ ≤ *c*_1_, then the problem (1) has at least two nontrivial solutions.

## 2. The Proof of Theorem 2

 In this section, we first introduce a compact embedding theory which is an extension of the classical Rellich-Kondrachov compactness theorem; see Xuan [13].

**Lemma 3**

Assume that *Ω* ∈ ℝ^*N*^ is an open bounded domain with *C* ^1^ boundary and 0 ∈ *Ω*, *N* ≥ 3, −*∞* < *a* < *pN*/(*N* − *p*). Then the embedding *W*_*a*_^1,*p*^(*Ω*)↪*L*_*α*_^*γ*^(*Ω*) is continuous if 1 < *γ* ≤ *Np*/(*N* − *p*) and 0 ≤ *α* ≤ (1 + *a*)*γ* + *N*(1 − *γ*/*p*) and is compact if 1 ≤ *γ* < *Np*/(*N* − *p*) and 0 ≤ *α* < (1 + *a*)*γ* + *N*(1 − *γ*/*p*).

The following lemma is called the Mountain Pass Theorem, see [14] (also see [15]).

**Lemma 4 (Mountain Pass Theorem)**

Let *X* be a real Banach space and *J* ∈ *C* ^1^(*X*, ℝ^1^) with *J*(0,0) = 0. Suppose that *J*(*u*, *v*) satisfies (*PS*) condition and

(*A*_1_) there are *ρ*, *α*_0_ > 0, such that *J*(*u*, *v*) ≥ *α*_0_ when ||(*u*,*v*)||_*X*_ = *ρ*;
(*A*_2_) there is (*e*_1_, *e*_2_) ∈ *X*, ||(*e*_1_,*e*_2_)||_*X*_ > *ρ* such that *J*(*e*_1_, *e*_2_) < 0.

Define

(12) $$\begin{array}{c} \Gamma =\left\{r\in C^{1}\left(\left[0,1\right],X\right)\;|\;r\left(0\right)=\left(0,0\right),r\left(1\right)=\left(e_{1},e_{2}\right)\right\}. \end{array}$$

Then

(13) $$\begin{array}{c} c=\underset{r\in \Gamma }{\inf}\;\underset{0\leq t\leq 1}{\max}\;J\left(r\left(t\right)\right)\geq \alpha_{0} \end{array}$$

is a critical value of *J*(*u*, *v*).

**Lemma 5**

Assume that (*H*_1_)–(*H*_3_) hold. There exist *c*_0_, *c*_1_ > 0 such that if the parameters *λ*, *μ* satisfy 0 < *λh*_1*θ*_ + *μh*_2*θ*_ < *c*_0_, and 0 ≤ *l*_1*σ*_^*p*/(*p*−1)^ + *l*_2*σ*_^*p*/(*p*−1)^ ≤ *c*_1_, then *J*_*λ*,*μ*_(*u*, *v*) satisfies the assumptions (*A*_1_)–(*A*_2_) in Lemma 4.

**Proof**

Let (*u*, *v*) ∈ *X*. By (8),

(14) Jλ,μ(u,v)=1p||(u,v)||p−1α+β∫ℝNg(x)|u|α|v|βdx−1γGλ,μ(u,v)−L(u,v).

By (*H*_2_) and the Hölder inequality, we have

(15) ∫ℝNg(x)|u|α+βdx≤(∫ℝN(g(x)|x|b(α+β))δdx)1/δ ×(∫ℝN|x|−bp∗|u|p∗dx)(α+β)/p∗≤gδSα+β||(u,v)||α+β,

where *g*_*δ*_ = (∫_ℝ^*N*^_(*g*(*x*) | *x*|^*b*(*α*+*β*)^)^*δ*^ *dx*)^1/*δ*^, *δ* = p*/(p* − *α* − *β*).

Similarly,

(16) $$\begin{array}{c} \int_{{\mathbb{R}}^{N}}g\left(x\right){\left|v\right|}^{\alpha +\beta }dx\leq g_{\delta }S^{\alpha +\beta }{||\left(u,v\right)||}^{\alpha +\beta }. \end{array}$$

Hence, it follows from (15) and (16) that

(17) $$\begin{array}{c} \int_{{\mathbb{R}}^{N}}g\left(x\right){\left|u\right|}^{\alpha }{\left|v\right|}^{\beta }dx\leq 2g_{\delta }S^{\alpha +\beta }{||\left(u,v\right)||}^{\alpha +\beta }. \end{array}$$

Since *h*_1_(*x*) | *x*|^*bγ*^ ∈ *L* ^*θ*^(ℝ^*N*^)∩*L* ^*∞*^(ℝ^*N*^), we obtain from the Hölder inequality and (5) that

(18) ∫ℝNλh1(x)|u|γdx≤λ(∫ℝN(h1(x)|x|bγ)θdx)1/θ ×(∫ℝN|x|−bp∗|u|p∗dx)γ/p∗≤λSγh1θ||(u,v)||γ.

Similarly,

(19) $$\begin{array}{c} \int_{{\mathbb{R}}^{N}}\mu h_{2}\left(x\right){\left|v\right|}^{\gamma }dx\leq \mu S^{\gamma }h_{2\theta }{||\left(u,v\right)||}^{\gamma }. \end{array}$$

Then,

(20) $$\begin{array}{c} G_{\lambda ,\mu }\left(u,v\right)\leq \left(\lambda h_{1\theta }+\mu h_{2\theta }\right)S^{\gamma }{||\left(u,v\right)||}^{\gamma }. \end{array}$$

Using the Hölder inequality, we get

(21) ∫ℝN|l1(x)||u|dx≤(∫ℝN|x|−bp∗|u|p∗dx)1/p∗   ×(∫ℝN|x|bσ|l1(x)|σdx)1/σ≤ε||(u,v)||p+Cε(Sl1σ)p/(p−1),

where *ε* > 0, *C*_*ε*_ > 0.

Similarly, we have

(22) ∫ℝN|l2(x)||v|dx≤Sl2σ||(u,v)||≤ε||(u,v)||p+Cε(Sl2σ)p/(p−1).

Thus,

(23) Jλ,μ(u,v)≥1p||(u,v)||p−1α+β·2gδSα+β||(u,v)||α+β −1γ(λh1θ+μh2θ)Sγ||(u,v)||γ−2ε||(u,v)||p −Cε(Sl1σ)p/(p−1)−Cε(Sl2σ)p/(p−1)≥12p||(u,v)||p−2gδα+βSα+β||(u,v)||α+β −1γ(λh1θ+μh2θ)Sγ||(u,v)||γ−Cε(Sl1σ)p/(p−1) −Cε(Sl2σ)p/(p−1)

with 0 < *ε* ≤ 1/4*p*.

Let

(24) $$\begin{array}{c} g\left(z\right)=\frac{2g_{\delta }}{\alpha +\beta }S^{\alpha +\beta }z^{\alpha +\beta -p}+\frac{1}{\gamma }\left(\lambda h_{1\theta }+\mu h_{2\theta }\right)S^{\gamma }z^{\gamma -p},\;z>0. \end{array}$$

To verify (*A*_1_) in Lemma 4, it suffices to show that *g*(*z*_1_) < 1/2*p* for some *z*_1_ = ||(*u*, *v*)|| > 0.

Note that *g*(*z*)→+*∞* where *z* → 0^+^ or *z* → +*∞*. Then *g*(*z*) has a minimum at *z*_1_ > 0. In order to find *z*_1_, we have

(25) g′(z)=2gδ(α+β−p)α+βSα+βzα+β−p−1+γ−pγ(λh1θ+μh2θ)Sγzγ−p−1.

So that *g*′(*z*_1_) = 0 and

(26) z1=((α+β)(p−γ)2γgδ(α+β−p)Sα+β−γ)1/(α+β−γ) ×(λh1θ+μh2θ)1/(α+β−γ)=c2(λh1θ+μh2θ)1/(α+β−γ)>0.

Moreover, *g*(*z*_1_) < 1/2*p* implies that

(27) g(z1)=(2gδα+βSα+βc2α+β−p+1γSγc2γ−p) ×(λh1θ+μh2θ)(α+β−p)/(α+β−γ)=c3(λh1θ+μh2θ)(α+β−p)/(α+β−γ)<12p,

where *c*_3_ = (2*g*_*δ*_/(*α* + *β*))*S* ^*α*+*β*^ *c*_2_^*α*+*β*−*p*^ + (1/*γ*)*S* ^*γ*^ *c*_2_^*γ*−*p*^.

Thus, it follows from (23) and (27) that there exist *c*_0_, *c*_1_, *α*_0_ > 0 such that *J*_*λ*,*μ*_(*u*, *v*) ≥ *α*_0_ with 0 < *λh*_1*θ*_ + *μh*_2*θ*_ < *c*_0_ and 0 ≤ *l*_1*σ*_^*p*/(*p*−1)^ + *l*_2*σ*_^*p*/(*p*−1)^ ≤ *c*_1_. Then *J*_*λ*,*μ*_(*u*, *v*) satisfies the assumption (*A*_1_) in Lemma 4.

We now verify (*A*_2_) in Lemma 4. Choose (*φ*_1_, *φ*_2_) ∈ *C*_0_^*∞*^(ℝ^*N*^)  ×  *C*_0_^*∞*^(ℝ^*N*^) such that

(28) $$\begin{array}{c} \int_{{\mathbb{R}}^{N}}g\left(x\right){\left|\varphi_{1}\right|}^{\alpha }{\left|\varphi_{2}\right|}^{\beta }dx>0. \end{array}$$

Then

(29) Jλ,μ(tφ1,tφ2)=tpp||(φ1,φ2)||p−tα+βα+β∫ℝNg(x)|φ1|α|φ2|βdx−tγγGλ,μ(φ1,φ2)−tL(φ1,φ2)

and *J*_*λ*,*μ*_(*tφ*_1_, *tφ*_2_)→−*∞* (*t* → +*∞*), since *α* + *β* > *p* > *γ*. Therefore, there exists *t* large enough, such that *J*_*λ*,*μ*_(*tφ*_1_, *tφ*_2_) < 0. Then we take *e* = (*tφ*_1_, *tφ*_2_) ∈ *X* and *J*_*λ*,*μ*_(*e*) < 0. The condition (*A*_2_) of Lemma 4 is true. This completes the proof of Lemma 5.

**Lemma 6**

Let (*H*_1_)-(*H*_2_) hold.

If {*u*_*n*_} and {*v*_*n*_} are bounded sequences in *W*_*a*_^1,*p*^(ℝ^*N*^), then there exist subsequences, still denoted {*u*_*n*_} and {*v*_*n*_}, and *u* ∈ *W*_*a*_^1,*p*^(ℝ^*N*^), *v* ∈ *W*_*a*_^1,*p*^(ℝ^*N*^) such that as *n* → *∞*

(30) $$\begin{array}{c} \overset{}{\underset{{\mathbb{R}}^{N}}{\int }}g\left(x\right){\left|u_{n}\right|}^{\alpha }dx\to \overset{}{\underset{{\mathbb{R}}^{N}}{\int }}g\left(x\right){\left|u\right|}^{\alpha }dx,\\ \overset{}{\underset{{\mathbb{R}}^{N}}{\int }}h_{1}\left(x\right){\left|u_{n}\right|}^{\gamma }dx\to \overset{}{\underset{{\mathbb{R}}^{N}}{\int }}h_{1}\left(x\right){\left|u\right|}^{\gamma }dx,\\ \overset{}{\underset{{\mathbb{R}}^{N}}{\int }}g\left(x\right){\left|v_{n}\right|}^{\alpha }dx\to \overset{}{\underset{{\mathbb{R}}^{N}}{\int }}g\left(x\right){\left|v\right|}^{\alpha }dx,\\ \overset{}{\underset{{\mathbb{R}}^{N}}{\int }}h_{2}\left(x\right){\left|v_{n}\right|}^{\gamma }dx\to \overset{}{\underset{{\mathbb{R}}^{N}}{\int }}h_{2}\left(x\right){\left|u\right|}^{\gamma }dx. \end{array}$$

**Proof**

Let *U*_*k*_ = {*x* ∈ ℝ^*N*^ | |*x* | <*k*}, *k* = 1,2,….

Since {*u*_*n*_} is bounded in *W*_*a*_^1,*p*^(ℝ^*N*^), {*u*_*n*_} is bounded in *W*_*a*_^1,*p*^(*U*_*k*_) for for all *k* ≥ 1. By Lemma 3, {*u*_*n*_} has a subsequence {*u*_*n*_^(1)^} which converges ${\overset{-}{u}}_{1}$ in *L* ^*γ*^(*U*_1_). Likewise, the subsequence {*u*_*n*_^(1)^} is bounded in *W*_*a*_^1,*p*^(*U*_2_). So that it has a subsequence {*u*_*n*_^(2)^} which converges ${\overset{-}{u}}_{2}$ in *L* ^*γ*^(*U*_2_). Since {*u*_*n*_^(2)^} is a subsequence of {*u*_*n*_^(1)^}, ${\overset{-}{u}}_{2}={\overset{-}{u}}_{1}$ in ${\bar{U}}_{1}$. Continuing this process, we obtain a sequence $\left\{{\overset{-}{u}}_{k}\right\}$ with the following properties:

(31) $$\begin{array}{c} {\overset{-}{u}}_{k}\in L^{\gamma }\left(U_{k}\right),\\ {\overset{-}{u}}_{k}\left(x\right)={\overset{-}{u}}_{1}\left(x\right),\;\text{a}.\text{e}\;\text{in}\;{\bar{U}}_{1},\\ {\overset{-}{u}}_{k}\left(x\right)={\overset{-}{u}}_{2}\left(x\right),\;\text{a}.\text{e}\;\text{in}\;{\bar{U}}_{2},\\ \vdots \\ {\overset{-}{u}}_{k}\left(x\right)={\overset{-}{u}}_{k-1}\left(x\right),\;\;\text{a}.\text{e}\;\text{in}\;{\bar{U}}_{k-1},\;k=1,2,\ldots . \end{array}$$

It is clear that ${\overset{-}{u}}_{k}(x)\to u(x)$ a.e in ℝ^1^, where

(32) $$\begin{array}{c} u\left(x\right)={\overset{-}{u}}_{k}\left(x\right),\;\;x\in {\bar{U}}_{k},\;\;k=1,2,\ldots . \end{array}$$

By a diagonal process, we take {*u*_*m*_^(*m*)^} which is a subsequence of {*u*_*n*_}. Thus, we have

(33) $$\begin{array}{c} u_{m}^{\left(m\right)}\to u,\;\text{strongly}\;\text{in}\;L^{\gamma }\left(U_{k}\right),\;k=1,2,\ldots . \end{array}$$

Without loss of generality, we assume that the subsequence {*u*_*m*_^(*m*)^} is {*u*_*n*_} itself. So

(34) $$\begin{array}{c} u_{n}\to u,\;\text{in}\;L^{\gamma }\left(U_{k}\right),\;k=1,2,\ldots . \end{array}$$

This implies that for *n* → *∞*,

(35) $$\begin{array}{c} u_{n}\left(x\right)\to u\left(x\right),\;\text{a}.\text{e}\;\text{in}\;U_{k},\;k=1,2,\ldots . \end{array}$$

Now, we prove

(36) $$\begin{array}{c} \underset{k\to \infty }{\lim}\underset{u\in W_{a}^{1,p}\setminus \left\{0\right\}}{\sup}\frac{{||u||}_{L^{\gamma }(U_{k}^{c},|h_{1}|)}}{{||u||}_{W_{a}^{1,p}({\mathbb{R}}^{N})}}=0. \end{array}$$

Indeed,

(37) ||u||Lγ(Ukc,|h1|)γ=∫Ukc|h1||u|γdx≤(∫Ukc(|h1||x|bγ)θdx)1/θ ×(∫Ukc|x|−bp∗|u|p∗dx)γ/p∗≤(∫Ukc(|h1||x|bγ)θdx)1/θSγ||u||Wa1,pγ,

where *θ* = p*/(p* − *γ*).

The fact *h*_1_(*x*) | *x*|^*bγ*^ ∈ *L* ^*θ*^(ℝ^*N*^) gives that lim⁡_*k*→*∞*_(∫_*U*_*k*_^*c*^_(|*h*_1_ | |*x*|^*bγ*^)^*θ*^ *dx*)^1/*θ*^ = 0.

Then, (37) implies that

(38)       ||u||Lγ(Ukc,|h1|)||u||Wa1,p≤S(∫Ukc(|h1||x|bγ)θdx)1/γθ→0,   as  k→∞.

This gives (36).

In the following, we show that

(39) $$\begin{array}{c} \int_{{\mathbb{R}}^{N}}h_{1}\left(x\right){\left|u_{n}\right|}^{\gamma }dx\to \int_{{\mathbb{R}}^{N}}h_{1}\left(x\right){\left|u\right|}^{\gamma }dx,\;\text{as}\;n\to \infty . \end{array}$$

Since the sequence {*u*_*n*_} is bounded in *W*_*a*_^1,*p*^(ℝ^*N*^), we can assume (up to a subsequence) that *u*_*n*_⇀*u* weakly in *W*_*a*_^1,*p*^(ℝ^*N*^) and ||*u*_*n*_||_*W*_*a*_^1,*p*^_ ≤ *C*_0_ for some constant *C*_0_ > 0 and all *n* ≥ 1.

By (36), we know that for any *ε* > 0, there exists *k*_*ε*_ > 0 so large that

(40) $$\begin{array}{c} {||u_{n}||}_{L^{\gamma }(U_{k_{\varepsilon }}^{c},|h_{1}|)}\leq C_{0}^{-1}\varepsilon {||u_{n}||}_{W_{a}^{1,p}}\leq \varepsilon \;\text{for}\;n=1,2,\ldots ,\\ {||u||}_{L^{\gamma }(U_{k_{\varepsilon }}^{c},|h_{1}|)}\leq \varepsilon . \end{array}$$

Since the embeddding *W*_*a*_^1,*p*^(*U*_*k*_*ε*__)↪*L* ^*γ*^(*U*_*k*_*ε*__) is compact (see Lemma 3), we have

(41) $$\begin{array}{c} \underset{n\to \infty }{\lim}{||u_{n}-u||}_{L^{\gamma }(U_{k_{\varepsilon }})}=0. \end{array}$$

Thus, there exists *N*_1_ > 0, when *n* > *N*_1_,

(42) $$\begin{array}{c} {||u_{n}-u||}_{L^{\gamma }(U_{k_{\varepsilon }})}<\varepsilon . \end{array}$$

So

(43) ||un−u||Lγ(ℝN,|h1|)≤||h1||L∞(ℝN)||un−u||Lγ(ℝN)≤||h1||L∞(ℝN)(||un||Lγ(Ukεc)+||u||Lγ(Ukεc)         +||un−u||Lγ(Ukε))≤3ε||h1||∞.

This shows that *u*_*n*_ → *u* in *L* ^*γ*^(ℝ^*N*^, |*h*_1_|) as *n* → *∞*.

By Brezis-Lieb's Lemma in [16], we obtain that (39) is true.

Similarly, we can prove

(44) $$\begin{array}{c} \int_{{\mathbb{R}}^{N}}g\left(x\right){\left|u_{n}\right|}^{\alpha }dx\to \int_{{\mathbb{R}}^{N}}g\left(x\right){\left|u\right|}^{\alpha }dx,\\ \int_{{\mathbb{R}}^{N}}g\left(x\right){\left|v_{n}\right|}^{\alpha }dx\to \int_{{\mathbb{R}}^{N}}g\left(x\right){\left|v\right|}^{\alpha }dx,\\ \int_{{\mathbb{R}}^{N}}h_{2}\left(x\right){\left|v_{n}\right|}^{\gamma }dx\to \int_{{\mathbb{R}}^{N}}h_{2}\left(x\right){\left|v\right|}^{\gamma }dx. \end{array}$$

**Remark 7**

By Brezis-Lieb's Lemma, it follows from (30) that

(45) $$\begin{array}{c} \int_{{\mathbb{R}}^{N}}\left|g\right|{\left|u_{n}-u\right|}^{\alpha }dx\to 0,\;\;\int_{{\mathbb{R}}^{N}}\left|g\right|{\left|v_{n}-v\right|}^{\alpha }dx\to 0,\\ \int_{{\mathbb{R}}^{N}}\left|h_{1}\right|{\left|u_{n}-u\right|}^{\gamma }dx\to 0,\;\;\int_{{\mathbb{R}}^{N}}\left|h_{2}\right|{\left|v_{n}-v\right|}^{\gamma }dx\to 0,\\ \int_{{\mathbb{R}}^{N}}\left|l_{1}\right|\left|u_{n}-u\right|dx\to 0,\;\;\int_{{\mathbb{R}}^{N}}\left|l_{2}\right|\left|v_{n}-v\right|dx\to 0. \end{array}$$

**Lemma 8**

Assume that (*H*_1_)–(*H*_3_) hold. Then *J*_*λ*,*μ*_(*u*, *v*) defined by (8) satisfies (*PS*) condition on *X*.

**Proof**

Let {(*u*_*n*_, *v*_*n*_)} be a (*PS*)_*c*_ sequence of *J*_*λ*,*μ*_(*u*, *v*) in *X*; that is, *J*_*λ*,*μ*_(*u*_*n*_, *v*_*n*_) → *c*, *J*_*λ*,*μ*_′(*u*_*n*_, *v*_*n*_) → 0, in X*. We first claim that {(*u*_*n*_, *v*_*n*_)} is bounded in *X*. In fact, for large *n*, we obtain

(46) c+1+||(un,vn)||≥Jλ,μ(un,vn) −1α+β〈Jλ,μ′(un,vn),(un,vn)〉=(1p−1α+β)||(un,vn)||p −(1γ−1α+β)Gλ,μ(un,vn) −(1−1α+β)L(un,vn).

Since *p* > *γ* > 1, we conclude that {(*u*_*n*_, *v*_*n*_)} is bounded. Then, the {*u*_*n*_} and {*v*_*n*_} are bounded in *W*_*a*_^1,*p*^(ℝ^*N*^), respectively. Thus, there exist {(*u*, *v*)} ∈ *X* and a subsequence of {(*u*_*n*_, *v*_*n*_)}, still denoted by {(*u*_*n*_, *v*_*n*_)}, such that

(47) $$\begin{array}{c} u_{n}⇀u,\;v_{n}⇀v\;\text{weakly}\;\text{in}\;W_{a}^{1,p}\left({\mathbb{R}}^{N}\right). \end{array}$$

Denote

(48) Pn=〈Jλ,μ′(un,vn),(un−u,vn−v)〉=∫ℝN(|x|−ap|∇un|p−2∇un∇(un−u)    +f1(x)|un|p−2un(un−u)    +|x|−ap|∇vn|p−2∇vn∇(vn−v)    +f2(x)|vn|p−2vn(vn−v))dx −αα+β∫ℝN(g(x)|un|α−2un|vn|β         +λh1(x)|un|γ−2un) ×(un−u)dx−βα+β ×∫ℝN(g(x)|vn|β−2vn|un|α      +μh2(x)|vn|γ−2vn)(vn−v)dx −∫ℝNl1(x)(un−u)dx −∫ℝNl2(x)(vn−v)dx.

Then the fact *J*_*λ*,*μ*_′(*u*_*n*_, *v*_*n*_) → 0 in X* implies *P*_*n*_ → 0 as *n* → *∞*.

Since that *u*_*n*_⇀*u*, *v*_*n*_⇀*v* in *W*_*a*_^1,*p*^(ℝ^*N*^), we see that

(49) Qn=∫ℝN(|x|−ap|∇u|p−2∇u∇(un−u)    +f1(x)|u|p−2u(un−u))dx +∫ℝN(|x|−ap|∇v|p−2∇v∇(vn−v)     +f2(x)|v|p−2v(vn−v))dx →0, n→∞.

Let

(50) Tn=∫ℝN(|x|−ap|∇un|p−2∇un∇(un−u)    +f1(x)|un|p−2un(un−u)    +|x|−ap|∇vn|p−2∇vn∇(vn−v)    +f2(x)|vn|p−2vn(vn−v))dx.

From dominated convergence theory and Lemma 6, we can conclude

(51) $$\begin{array}{c} \int_{{\mathbb{R}}^{N}}g\left(x\right){\left|u_{n}\right|}^{\alpha -2}u_{n}{\left|v_{n}\right|}^{\beta }\left(u_{n}-u\right)dx\to 0,\;n\to \infty , \end{array}$$

(52) $$\begin{array}{c} \int_{{\mathbb{R}}^{N}}g\left(x\right){\left|v_{n}\right|}^{\beta -2}v_{n}{\left|u_{n}\right|}^{\alpha }\left(v_{n}-v\right)dx\to 0,\;n\to \infty . \end{array}$$

From Hölder inequality,

(53) ∫ℝN|h1(x)||un|γ−1|un−u|dx≤(∫ℝN|h1||un−u|γdx)1/γ ×(∫ℝN|h1||un|γdx)(γ−1)/γ→0, as  n→∞.

Similarly, we have

(54) $$\begin{array}{c} \int_{{\mathbb{R}}^{N}}\left|h_{2}\right|{\left|v_{n}\right|}^{\gamma -1}\left|v_{n}-v\right|dx\to 0,\\ \int_{{\mathbb{R}}^{N}}\left|l_{1}\left(x\right)\right|\left|u_{n}-u\right|dx\to 0,\\ \int_{{\mathbb{R}}^{N}}\left|l_{2}\left(x\right)\right|\left|v_{n}-v\right|dx\to 0. \end{array}$$

Then it follows from (48), (49) to (54) that *T*_*n*_ → 0, as *n* → *∞*.

So

(55) Tn−Qn=∫ℝN|x|−ap(|∇un|p−2∇un      −|∇u|p−2∇u)∇(un−u)dx+∫ℝNf1(x)(|un|p−2un−|u|p−2u)(un−u)dx+∫ℝN|x|−ap(|∇vn|p−2∇vn       −|∇v|p−2∇v)∇(vn−v)dx+∫ℝNf2(x)(|vn|p−2vn−|v|p−2v)  ×(vn−v)dx→0.

Using the standard inequality in ℝ^*N*^ given by

(56) 〈|ξ|p−2ξ−|η|p−2η,ξ−η〉≥Cp|ξ−η|p, p≥2,〈|ξ|p−2ξ−|η|p−2η,ξ−η〉≥Cp|ξ−η|2(|ξ|+|η|)p−2,1<p<2,

we have from (55) that ||(*u*_*n*_ − *u*, *v*_*n*_ − *v*)|| → 0, as *n* → *∞*.

Thus, *J*_*λ*,*μ*_(*u*, *v*) satisfies (*PS*)_*c*_ condition on *X*.

**Proof of Theorem 2**

By Lemmas 5 and 8, *J*_*λ*,*μ*_(*u*, *v*) satisfies all assumptions in Lemma 4. Then there exists (*u*_1_, *v*_1_) ∈ *X* such that (*u*_1_, *v*_1_) is a solution of problem (1) by Lemma 4. Furthermore, *J*_*λ*,*μ*_(*u*_1_, *v*_1_) ≥ *α*_0_ > 0.

We now seek a solution (*u*_2_, *v*_2_) of problem (1). Choose (*φ*_1_, *φ*_2_) ∈ *C*_0_^*∞*^(ℝ^*N*^) × *C*_0_^*∞*^(ℝ^*N*^) such that ∫_ℝ^*N*^_ *l*_1_(*x*)*φ*_1_ *dx* + ∫_ℝ^*N*^_ *l*_2_(*x*)*φ*_2_ *dx* > 0, and then

(57) Jλ,μ(tφ1,tφ2)=1ptp||(φ1,φ2)||p−1α+βtα+β∫ℝNg(x)|φ1|α|φ2|βdx−t∫ℝN(l1(x)φ1+l2(x)φ2)dx−1γtγGλ,μ(φ1,φ2)<0

for small *t* > 0 and thus for any open ball *B*_*τ*_ ⊂ *X* such that

(58) $$\begin{array}{c} -\infty <c_{\tau }=\underset{{\bar{B}}_{\tau }}{\inf}\;J_{\lambda ,\mu }\left(u,v\right)<0. \end{array}$$

Thus, exists *ρ* > 0, such that

(59) $$\begin{array}{c} c_{\rho }=\underset{\left(u,v\right)\in {\bar{B}}_{\rho }}{\inf}J_{\lambda ,\mu }\left(u,v\right)<0,\;\;\underset{\left(u,v\right)\in \partial B_{\rho }}{\inf}J_{\lambda ,\mu }\left(u,v\right)>0. \end{array}$$

Letting *ε*_*n*_ ↓ 0 such that

(60) $$\begin{array}{c} 0<\varepsilon_{n}<\underset{\left(u,v\right)\in \partial B_{\rho }}{\inf}J_{\lambda ,\mu }\left(u,v\right)-\underset{\left(u,v\right)\in B_{\rho }}{\inf}J_{\lambda ,\mu }\left(u,v\right). \end{array}$$

Then, by Ekeland's variational principle in [17], there exists $\{(u_{n},v_{n})\}\subset {\bar{B}}_{\rho }$ such that

(61) $$\begin{array}{c} c_{\rho }\leq J_{\lambda ,\mu }\left(u_{n},v_{n}\right)<c_{\rho }+\varepsilon_{n}, \end{array}$$

(62) Jλ,μ(un,vn)<Jλ,μ(u,v)+εn||(un−u,vn−v)||,   ∀(u,v)∈B¯ρ,  u≠un,  v≠vn.

Then it follows from (59) to (61) that

(63) Jλ,μ(un,vn)<cρ+εn≤inf⁡(u,v)∈BρJλ,μ(u,v)+εn<inf⁡(u,v)∈∂BρJλ,μ(u,v),

so that (*u*_*n*_, *v*_*n*_) ∈ *B*_*ρ*_. We now consider the functional $F:{\bar{B}}_{\rho }\to \mathbb{R}$ given by

(64) $$\begin{array}{c} F\left(u,v\right)=J_{\lambda ,\mu }\left(u,v\right)+\varepsilon_{n}||\left(u-u_{n},v-v_{n}\right)||,\;\left(u,v\right)\in {\bar{B}}_{\rho }. \end{array}$$

Then (62) shows that *F*(*u*_*n*_, *v*_*n*_) < *F*(*u*, *v*), $(u,v)\in {\bar{B}}_{\rho }$, *u* ≠ *u*_*n*_, *v* ≠ *v*_*n*_, and thus (*u*_*n*_, *v*_*n*_) is a strict local minimum of *F*. Moreover,

(65) t−1(F(un+tφ1,vn+tφ2)−F(un,vn))≥0, for  small  t>0,∀(φ1,φ2)∈B1.

Hence,

(66) t−1(Jλ,μ(un+tφ1,vn+tφ2)−Jλ,μ(un,vn))  +εn||(φ1,φ2)||≥0.

Let *t* → 0^+^; then,

(67) 〈Jλ,μ′(un,vn),(φ1,φ2)〉+εn||(φ1,φ2)||≥0,    ∀(φ1,φ2)∈B1.

Replacing (*φ*_1_, *φ*_2_) in (67) by (−*φ*_1_, −*φ*_2_), we get

(68) −〈Jλ,μ′(un,vn),(φ1,φ2)〉+εn||(φ1,φ2)||≥0,    ∀(φ1,φ2)∈B1.

So that ||*J*_*λ*,*μ*_′(*u*_*n*_, *v*_*n*_)|| ≤ *ε*_*n*_.

Therefore, there is a sequence {(*u*_*n*_, *v*_*n*_)} ⊂ *B*_*ρ*_ such that *J*_*λ*,*μ*_(*u*_*n*_, *v*_*n*_) → *c*_*ρ*_ < 0, and *J*_*λ*,*μ*_′(*u*_*n*_, *v*_*n*_) → 0 in X* as *n* → *∞*. By Lemma 5, {(*u*_*n*_, *v*_*n*_)} has a convergent subsequence in *X*, still denoted by {(*u*_*n*_, *v*_*n*_)}, such that (*u*_*n*_, *v*_*n*_)→(*u*_2_, *v*_2_) in *X*. Thus (*u*_2_, *v*_2_) is a solution of (1) with *J*_*λ*,*μ*_(*u*_2_, *v*_2_) < 0. Then the proof of Theorem 2 is complete.
